# Safety and tolerability of topical trametinib in rosacea: Results from a phase I clinical trial

**DOI:** 10.1002/ski2.346

**Published:** 2024-01-31

**Authors:** Edward J. Wladis, Jacqueline Busingye, Leahruth K. Saavedra, Amy Murdico, Alejandro P. Adam

**Affiliations:** ^1^ Department of Ophthalmology Lions Eye Institute Albany Medical College Albany New York USA; ^2^ Department of Ophthalmology Albany Stratton Veterans Affairs Medical Center Albany New York USA; ^3^ Department of Otolaryngology Albany Medical College Albany New York USA; ^4^ Department of Molecular and Cellular Physiology Albany Medical College Albany New York USA

## Abstract

**Purpose:**

Overactivation of the mitogen activated kinase pathway has been associated with rosacea. We hypothesised that inhibitors of this pathway can be repurposed to alleviate rosacea symptoms.

**Methods:**

In order to test this hypothesis, we designed a double‐blind, randomised, placebo‐controlled phase I clinical trial to assess the safety and tolerability of a first‐in‐kind topical formulation of a MEK kinase inhibitor, trametinib. Subjects applied daily trametinib‐containing cream (0.05 mg in 0.5 mL) to one cheek and cream without inhibitor to the other for consecutive 21 days. Skin irritation scores and blood samples were obtained during visits on days 8, 15 and 22.

**Results:**

On analysis of high‐performance liquid chromatography, no systemic trametinib absorption was detected during this treatment period. Subjects demonstrated a slight but significant improvement in both cheeks, regardless of drug contents. No adverse effects were reported during this time.

**Conclusions:**

Topical trametinib was well tolerated at a dose of 0.05 mg per day without meaningful systemic absorption or local adverse events. A dose escalation trial is warranted to determine optimal dosing to treat rosacea while avoiding the adverse effects of systemic treatment.

## Graphical abstract

Preclinical investigations have implicated particular kinases in the pathogenesis of rosacea, suggesting that interference with these proteins may be an effective treatment strategy to address these disease. This phase I trial tested a topical kinase inhibitor to treat rosacea, and the medication was found to be very safe. These results suggest that this medication is a good candidate for later‐stage testing.

**What is already known about this topic?**

- p38 and ERK are enriched in cutaneous specimens of rosacea, and a variety of cellular features suggest that interference with the MAP kinase might be an effective treatment strategy to address rosacea.

**What does this study add?**

- This study demonstrates that a medication to suppress MAP kinase can be delivered as a topical preparation. This therapy was uniformly safe and the medication was not absorbed systemically. As such, this therapeutic approach can safely be tested for efficacy in later‐stage trials.

## 1 INTRODUCTION

Approximately 10% of the population of the United States suffers from rosacea,^1^ resulting in considerable cutaneous inflammation,^2^ interference with activities of daily living, and psychosocial burden.^3^, ^4^, ^5^, ^6^ Despite this high burden associated with of rosacea,^7^ current therapies for this disorder are generally ineffective and associated with significant side effects.^8^ Historically, a limited understanding of this disease prevented the design of medications specifically targeted to rosacea vpathophysiology.

Recent work provided new insights into the biology of the disease by identifying discrete activation of mitogen‐associated protein kinase (mitogen activated kinase pathway [MAPK]) signalling governing its cutaneous inflammation, thus raising hopes for highly targeted therapeutic interventions. Previously, Wladis et al identified increased activation of p38 and ERK in cutaneous specimens of rosacea.^9^ Similarly, Harden et al demonstrated upregulation of MAPK signalling in explants of papulopustular skin specimens.^10^ Kan et al reported that cinnamtannin B1 suppressed the cutaneous inflammation of rosacea in a mouse model through inhibition of ERK phosphorylation.^11^ Additionally, several lines of evidence indicate that inhibition of MAPK results in in vitro suppression of inflammatory mediators in a variety of other conditions.^12^, ^13^, ^14^, ^15^ We have previously shown that trametinib limits the acute increases in endothelial permeability in response to the pro‐inflammatory cytokine IL‐6.^16^ Together, these findings suggest the inhibition of MAPK may selectively arrest the inflammation associated with rosacea in a specific fashion, thereby providing a meaningful interference of the disease with fewer complications.

The canonical MAPK signalling pathway involves the activation of ERK1 and ERK2 via phosphorylation by MEK1 and MEK2 kinases. Many highly specific inhibitors of this pathway have been developed, with several inhibitors (trametinib, binimetinib, selumetinib, and cobimetinib) being approved by the United States' Food and Drug Administration (FDA) for the treatment of specific cancers.^17^, ^18^ We thus hypothesised that these drugs could be repurposed for the treatment of rosacea and other inflammatory cutaneous diseases. This trial was performed to assess the safety and tolerability of topical trametinib in patients with rosacea. This approach was employed to interfere with the previously implicated signalling activity that subserves rosacea in a fashion that would minimise systemic absorption and therefore avoid the adverse effects associated with oral intake.

## 2 RESULTS

**FIGURE 1:**
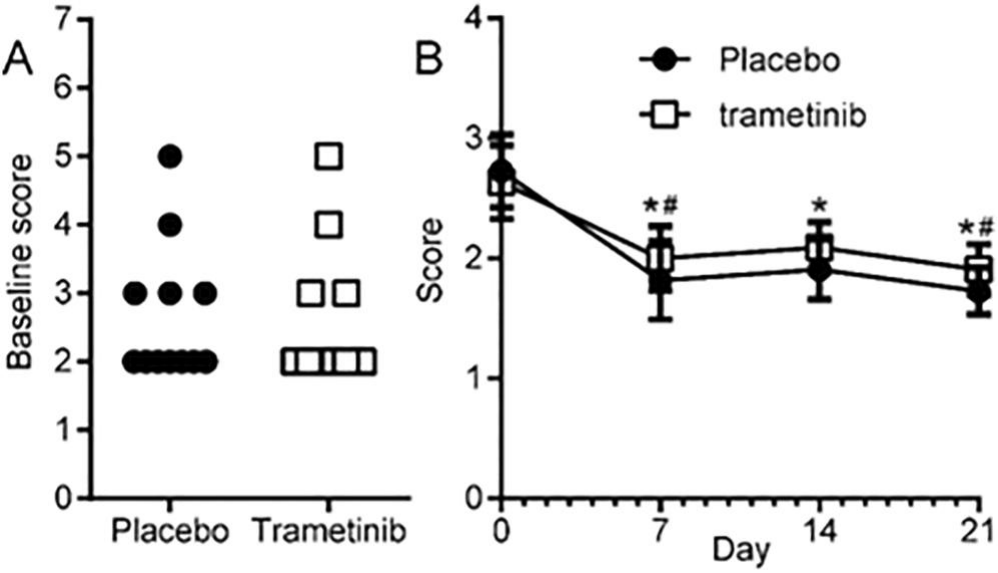
Skin irritation scoring system scores. (a) baseline scores in each cheek, separated by stratification. No significant differences were observed (*p* > 0.8, Mann–Whitney). (b) Mean + SEM scores in each cheek. Significant differences with time, but not between treatments. *, placebo *p* < 0.05 versus day 0. #, *p* < 0.05 trametinib versus day 0. Two‐way ANOVA of repeated measurements and Holm‐Sidak post‐hoc test.

Twelve patients were identified during the recruitment period. The characteristics of these patients are summarised in Table 1. All patients completed the study, and no patient withdrew from the trial for reasons of adverse events or intolerance of the medication. One patient missed a study visit, due to travel. No statistically significant differences were observed on the baseline scores between the assigned treated and untreated sides (Figure 1a).

**TABLE 1:** Patient demographic information and Skin Irritation Scoring System scores.

Patient ID					SISS score at day			
	Age	Gender	Race/Ethnicity	Side	Baseline	7	14	21
1	76	Male	Caucasian	Placebo	4	4	4	2
				Trametinib	4	4	4	2
2	67	Male	Caucasian	Placebo	3	2	2	2
				Trametinib	3	2	2	2
3	63	Male	Caucasian	Placebo	3	3	2	2
				Trametinib	3	2	2	2
4	72	Male	Caucasian	Placebo	5	2	2	2
				Trametinib	5	2	2	2
5	68	Male	Caucasian	Placebo	2	0	1	1
				Trametinib	2	1	2	1
6	74	Male	Caucasian	Placebo	2	1	2	1
				Trametinib	2	3	2	3
7	74	Male	Caucasian	Placebo	2	1	2	2
				Trametinib	2	1	2	2
8	63	Male	Caucasian	Placebo	2	2	1	1
				Trametinib	2	2	2	1
9	74	Male	Caucasian	Placebo	3	2	2	2
				Trametinib	2	1	2	2
10	58	Male	Caucasian	Placebo	2	1	1	1
				Trametinib	2	2	1	1
11	67	Male	Caucasian	Placebo	2	2	2	3
				Trametinib	2	2	2	3
12	63	Male	Caucasian	Placebo	2	2	NA	2
				Trametinib	2	2	NA	2

The two sides of the face were not statistically significantly different from one another at any time point. We noticed a slight reduction in the Skin Irritation Scoring System (SISS)^19^ scores on both sides, which we attributed to a mild effect of applying the ointment every day (Table 1 and Figure 1b).

Levels of trametinib in blood remained below the limit of detection by high‐performance liquid chromatography (HPLC) (<0.1 ng/mL) as measured from a blood sample obtained at every visit. At the end of the trials the bottles were returned for weighing. Two subjects did not return the bottles. The average weight loss of each bottle was 5.45 g. No statistically significant difference was observed between the placebo and trametinib bottles (5.64 ± 2.14 g vs. 5.26 ± 2.16 g).

## 3 DISCUSSION

Multiple therapeutic options are routinely employed to address rosacea, because each modality suffers from incomplete responses and adverse events.^8^ Oral and topical antibiotics, lifestyle modification, immunomodulatory agents, and laser‐ and light‐based therapies are commonly used to treat this disorder, although the literature indicates limited utility, considerable commitment from patients, frustration with outcomes, lack of adherence, and serious side effects.^20^, ^21^, ^22^, ^23^, ^24^

Several studies have explored the pathogenesis of rosacea, and our understanding of the cutaneous biology of the disease has evolved considerably.^25^ Specifically, several biologic aberrancies appear to contribute to the development of rosacea, including toll‐like receptors,^26^, ^27^ kallikrein‐related peptidase 5 and cathelicidin LL‐37,^28^ nuclear factor κB,^29^ myeloid differential factor‐88,^30^ and multiple genetic factors.^31^, ^32^ These steps towards a pathogenetic construct suggest multiple discrete checkpoints that may be amenable to selective manipulation for the treatment of rosacea.

Wladis et al previously implicated p38 and ERK in the pathogenesis of rosacea,^9^ suggesting a role for suppression of the MAPK pathway to arrest rosacea. MAPK inhibition has emerged as a meaningful treatment strategy for several diseases, including melanoma, non‐small cell lung cancer, and neurofibromatosis.^33^, ^34^, ^35^ The current study repurposed an FDA‐approved MEK inhibitor as a topical preparation to target rosacea. To minimise systemic absorption, we suspended trametinib in a highly hydrophobic base. Consistent with minimal absorption, we did not detect any drug in circulation, indicating that patients are very unlikely to experience systemic adverse events. Furthermore, we did not detect any local adverse events, and the active medication was well‐tolerated. It is possible that absorption in other skin types may be different. However, rosacea is highly prevalent in populations with fair skin and older,^36^, ^37^ and thus this finding remains clinically relevant. Based on these findings, topical trametinib represents a scientifically‐sound approach to combat rosacea in a highly targeted fashion without inducing the side effects that are classically associated with our current therapeutic armamentarium.

This important proof of principle raises significant questions for future study. Specifically, while this phase one trial underscored the safety and tolerability of MAPK inhibition in patients that suffer from rosacea, the investigation was not specifically powered or of adequate duration to assess the efficacy of this approach. Due to the characteristics of the population at the recruiting center, all patients enroled were Caucasian males. Alternative centers will be required to expand the enrolment to females and other races and ethnicities. Additionally, only one dose of the medication was employed in this early trial. A dose‐escalating phase I/II trial must be implemented to determine the optimal concentration of the medication to control the disease and avoid local and systemic toxicities. A modest but significant decrease in scoring was observed in both cheeks. We attributed this improvement to the daily care on skin that otherwise may not have received any cream. Alternatively, this effect could be due to improve adherence to the current care, a potential example of the Hawthorne effect.^38^, ^39^, ^40^ Trials designed to test for efficacy in the absence of other skin treatments will be required to better understand this finding. We do not believe this could be due to the effect of the contralateral trametinib application since this would require systemic absorption and trametinib plasma levels remained below the level of detection. It remains unknown if this effect would disappear after prolonged application. Lastly, we cannot discard the possibility that the subjects applied the cream to opposite sides, masking any potential effect. Consequently, future research will need to explore the benefits of MAPK inhibitors and the ideal dosing of this topical preparation.

Several limitations apply to this investigation. Given that this study was designed to explore safety and tolerability, patients were allowed to continue their current treatments for rosacea. Similarly, the pharmacokinetics and dermal penetration will need to be carefully assessed. Lastly, this trial had a short duration and a limited number of subjects consisting of older Causasian males. While a longer trial of this therapeutic approach may uncover local toxicities, the 22‐day duration of this trial was chosen based on the guidelines of the FDA. Additionally, the absence of systemic absorption of the topical medication provides reassurance that systemic side effects are unlikely. Current pharmacological approaches require many months of treatment to achieve a clinically meaningful improvement. Our short duration trial was not designed to test the potential effect of longer treatments. The results of this trial provide opportunities to perform a dose‐escalating phase I/II trial on a more diverse subject population, and, at that point, rosacea therapies should be discontinued to assess the efficacy of MAPK inhibition.

Nonetheless, this study represents a very meaningful advance in our ability to provide targeted therapy for rosacea. Given that this therapeutic approach directly stems from improvements in our understanding of the cutaneous biology of the disease and that this safety study did not detect any adverse events, additional investigations of this very promising technology may be safely performed to provide relief to patients that suffer from this illness.

## 4 MATERIALS AND METHODS

### 4.1 Study design

This is a placebo self‐controlled, double‐blinded, randomised interventional study to assess safety and tolerability of a topical medication. This study was approved by the Institutional Review Board of the institution. It adhered to the tenets of the Declaration of Helsinki and the Health Insurance Portability and Accountability Act. The phase one trial was registered at clinicaltrials.gov.

## 5 Drug formulation

Extraction of trametinib from commercially available tablets and formulation on a hydrophobic Humco Anhydrous Base cat# 6013‐01 (Humco) was done following good laboratory practices manufacturing by a FDA‐registered compounding pharmacy (Pharmaceutic Labs, Colonie). An analytical test method was developed and validated by Biomed Innova (Albany, NY) for assaying Trametinib topical cream that was used in the study. It was analysed and quantified by HPLC. The results were consistent with the theoretical amount of 0.1 mg/g Trametinib and was therefore approved for the use in this study. All bottles dispensed to the subjects were weighted before and at the end of the trial.

### 5.1 Patient recruitment

Patients with erythematotelangiectatic rosacea were identified through the ophthalmology clinic, based on clinical examination. In order to be included, patients were required to meet the standard requirements for a diagnosis of rosacea, including central facial erythema with associated telangiectasias, transient erythema, dry eye disease, or phymomas. Patients were allowed to continue with their current regimens for the treatment of rosacea, although no patient was actively taking a medication to address rosacea at the time of recruitment. Exclusion criteria included age less than 18 and over 80 years, a documented allergy to trametinib, a concurrent condition that would preclude the use of trametinib, pregnancy, heart failure, and the concomitant use of medications that would interact unfavourably with trametinib. Upon identification, informed consent was obtained by the study coordinator. No restrictions were placed on sex, gender, race or ethnicity. Despite the broad criteria, all 12 enroled subjects were Caucasian males, reflecting the majority of the population at the center. All subjects received instruction in person by the investigators at the time of enrolment.

### 5.2 Study protocol

Medication was dispensed via vials at a controlled pharmacy. Specifically, patients received two 0.5 mL dispensing bottles that were labelled for either the right or left cheek. One container dispensed a suspension vehicle and served as a placebo control. The other bottle contained the same vehicle with 0.01% trametinib. In both cases, the suspension was made on a hydrophobic base to deliver the topical medication while minimising systemic absorption of the drug. The total amount delivered (0.05 mg/day) was determined by dividing the recommended dose of oral trametinib (2 mg/day) by an estimation of the total area to be covered (∼1% of the total body surface area). An excess material was delivered to account for cream loss and drug binding to stratum corneum and dead keratinocytes.

Through a computerised random number generator, patients were assigned to apply the contents of the placebo vial to one side of the face and the active medication to the contralateral side once each day over a 22‐day period, as suggested by FDA guidance.^19^ Local skin irritancy was assessed through the SISS on days 1, 8, 15, and 22 of the trial by an observer who was blinded as to which side received the active medication. Briefly, this test analyzes the development of erythema and oedema via a 0–7 scoring mechanism for skin appearance and additional effects ranging from 0 to 3.^19^ The scoring criteria is shown in Table 2.

**TABLE 2:** Skin appearance scoring system.

Skin appearance	Score
No evidence of irritation	0
Minimal erythema, barely perceptible	1
Definite erythema, readily visible; minimal oedema or minimal papular response	2
Erythema and papules	3
Definite oedema	4
Erythema, oedema, and papules	5
Vesicular eruption	6
Strong reaction spreading beyond test site	7

Other effects	Score	Numerical equivalent
Slight glazed appearance	A	0
Marked glazing	B	1
Glazing with peeling and cracking	C	2
Glazing with fissures	F	3
Film of dried serous exudate covering all or part of the patch site	G	3
Small petechial erosions and/or scabs	H	3

*Note*: When one or more ‘other effects’ are observed, the score should be reported as a dermal response number, a letter combination score, and as a numerical total (i.e., numerical ‘dermal response’ score + numeric equivalent for the ‘other effects’ lettered score). For example, the dermal response of 6 with glazing with fissure (F [3]) will equal to the score of 9. When no ‘other effects’ are observed, score zero should be applied to an observed ‘other effects’. Slightly modified from: Assessing the Irritation and Sensitisation Potential of Transdermal and Topical Delivery Systems for ANDAs. Guidance for Industry. DRAFT GUIDANCE. U.S. Department of Health and Human Services, Food and Drug Administration, Center for Drug Evaluation and Research (CDER), October 2018.^19^

During each visit (days 1, 8, 15, and 22), blood samples (5 mL) from the median cubital vein were collected in EDTA‐coated tubes and photographs of the treated areas were taken with a dedicated digital camera. All subjects declared following the procedures when asked at each visit.

### 5.3 Determination of trametinib in plasma

Plasma was obtained from each blood sample by standard centrifugation (1800 × g for 18 min at room temperature) and maintained at −20 C until use. Trametinib levels were measured trametinib levels were assayed via HPLC by Biomed Innova (Albany, NY). The minimum level of detection was determined to be 0.1 ng/mL by spiking pure trametinib (Selleck Chemicals) in normal plasma (Human Plasma with K3‐EDTA obtained from Avantor).

### 5.4 Statistical analysis

Direct comparisons of the SISS scores (Table 1) at each time were performed via a dedicated statistical software package (GraphPad Prism). Baseline levels in treated versus untreated sides were compared by Mann‐Whitney *U* test. The effect during each visit was compared via two‐way ANOVA of repeated measurements. *p*‐values of <0.05 were considered statistically significant.

## CONFLICT OF INTEREST STATEMENT

EJW and APA are co‐founders of Praxis Biotechnology, Inc. This company are inventors of and licensed an awarded patent (US‐20190142831‐A1) from Albany Medical College.

## AUTHOR CONTRIBUTIONS

**Edward J. Wladis**: Conceptualization (equal); formal analysis (equal); investigation (equal); methodology (equal); supervision (equal); validation (equal); writing – original draft (equal); writing – review & editing (equal). **Jacqueline Busingye**: Data curation (equal); investigation; methodology; resources (equal); writing – review & editing. **Leahruth K. Saavedra**: Data curation (equal); methodology; resources (equal); validation. **Amy Murdico**: Methodology; project administration (equal); resources (equal); validation; writing – review  & editing. **Alejandro P Adam**: Conceptualization. data curation (equal); formal analysis; investigation; methodology; project administration (equal); software (equal); supervision; validation; writing – review & editing.

## ETHICS STATEMENT

This study was approved by the institutional review board of the Albany Stratton Veterans Administration medical center.

## Data Availability

All available data is provided within this manuscript.

## References

[1] Tan J , Berg M . Rosacea: current state of epidemiology. J Am Acad Dermatol. 2013;69(6 Suppl 1):S27–S35. 10.1016/j.jaad.2013.04.043 24229634

[2] Bakar O , Demircay Z , Toker E , Cakir S . Ocular signs, symptoms and tear function tests of papulopustular rosacea patients receiving azithromycin. J Eur Acad Dermatol Venereol. 2009;23(5):544–549. 10.1111/j.1468-3083.2009.03132.x 19250326

[3] Bewley A , Fowler J , Schofer H , Kerrouche N , Rives V . Erythema of rosacea impairs health‐related quality of life: results of a meta‐analysis. Dermatol Ther. 2016;6(2):237–247. 10.1007/s13555-016-0106-9 PMC4906100 26983751

[4] Dirschka T , Micali G , Papadopoulos L , Tan J , Layton A , Moore S . Perceptions on the psychological impact of facial erythema associated with rosacea: results of international survey. Dermatol Ther. 2015;5(2):117–127. 10.1007/s13555-015-0077-2 PMC4470961 26022994

[5] Elewski BE . Results of a national rosacea patient survey: common issues that concern rosacea sufferers. J drugs dermatol JDD. 2009;8(2):120–123. 19213226

[6] Huynh TT . Burden of disease: the psychosocial impact of rosacea on a patient's quality of life. Am Health Drug Benefits. 2013;6(6):348–354. 24991368 PMC4031723

[7] Lim HW , Collins SAB , Resneck JS, Jr. , Bolognia JL , Hodge JA , Rohrer TA , et al. The burden of skin disease in the United States. J Am Acad Dermatol. 2017;76(5):958–72e2. 10.1016/j.jaad.2016.12.043 28259441

[8] Wladis EJ , Adam AP . Treatment of ocular rosacea. Surv Ophthalmol. 2017;63(3):340–346. 10.1016/j.survophthal.2017.07.005 28782548

[9] Wladis EJ , Swamy S , Herrmann A , Yang J , Carlson JA , Adam AP . Activation of p38 and erk mitogen‐activated protein kinases signaling in ocular rosacea. Invest Ophthalmol Vis Sci. 2017;58(2):843–848. 10.1167/iovs.16-20275 28170535

[10] Harden JL , Shih YH , Xu J , Li R , Rajendran D , Hofland H , et al. Paired transcriptomic and proteomic analysis implicates IL‐1beta in the pathogenesis of papulopustular rosacea explants. J Invest Dermatol. 2021;141(4):800–809. 10.1016/j.jid.2020.08.013 32941918

[11] Kan HL , Wang CC , Cheng YH , Yang CL , Chang HS , Chen IS , et al. Cinnamtannin B1 attenuates rosacea‐like signs via inhibition of pro‐inflammatory cytokine production and down‐regulation of the MAPK pathway. PeerJ. 2020;8:e10548. 10.7717/peerj.10548 33391878 PMC7759128

[12] Carter AB , Monick MM , Hunninghake GW . Both Erk and p38 kinases are necessary for cytokine gene transcription. Am J Respir Cell Mol Biol. 1999;20(4):751–758. 10.1165/ajrcmb.20.4.3420 10101008

[13] de Souza AP , Vale VL , Silva Mda C , Araujo IB , Trindade SC , de Moura‐Costa LF , et al. MAPK involvement in cytokine production in response to Corynebacterium pseudotuberculosis infection. BMC Microbiol. 2014;14(1):230. 10.1186/s12866-014-0230-6 25179342 PMC4167526

[14] Lucas RM , Luo L , Stow JL . ERK1/2 in immune signalling. Biochem Soc Trans. 2022;50(5):1341–1352. 10.1042/bst20220271 36281999 PMC9704528

[15] Scherle PA , Jones EA , Favata MF , Daulerio AJ , Covington MB , Nurnberg SA , et al. Inhibition of MAP kinase kinase prevents cytokine and prostaglandin E2 production in lipopolysaccharide‐stimulated monocytes. J Immunol. 1998;161(10):5681–5686. 10.4049/jimmunol.161.10.5681 9820549

[16] Alsaffar H , Martino N , Garrett JP , Adam AP . Interleukin‐6 promotes a sustained loss of endothelial barrier function via Janus kinase‐mediated STAT3 phosphorylation and de novo protein synthesis. Am J Physiol Cell Physiol. 2018;314(5):C589–C602. 10.1152/ajpcell.00235.2017 29351406

[17] Klomp JE , Klomp JA , Der CJ . The ERK mitogen‐activated protein kinase signaling network: the final frontier in RAS signal transduction. Biochem Soc Trans. 2021;49(1):253–267. 10.1042/bst20200507 33544118 PMC12691160

[18] Moore AR , Rosenberg SC , McCormick F , Malek S . RAS‐targeted therapies: is the undruggable drugged? Nat Rev Drug Discov. 2020;19(8):533–552. 10.1038/s41573-020-0068-6 32528145 PMC7809886

[19] FDA . Assessing the irritation and sensitization potential of transdermal and topical delivery systems for ANDAs: draft guidance for industry. Food & Drug Administration Center for Drug Evaluation and Research (CDER); 2018.

[20] Cai Y , Zhu Y , Wang Y , Xiang W . Intense pulsed light treatment for inflammatory skin diseases: a review. Laser Med Sci. 2022;37(8):3085–3105. 10.1007/s10103-022-03620-1 35913536

[21] Delans K , Kelly K , Feldman SR . Treatment strategies, including antibiotics, to target the immune component of rosacea. Expet Rev Clin Immunol. 2022;18(12):1239–1251. 10.1080/1744666x.2022.2128334 36137266

[22] van Zuuren EJ , Arents BWM , van der Linden MMD , Vermeulen S , Fedorowicz Z , Tan J . Rosacea: new concepts in classification and treatment. Am J Clin Dermatol. 2021;22(4):457–465. 10.1007/s40257-021-00595-7 33759078 PMC8200341

[23] van Zuuren EJ , Fedorowicz Z , Carter B , van der Linden MM , Charland L . Interventions for rosacea. Cochrane Database Syst Rev. 2015;2015(4):CD003262. 25919144 10.1002/14651858.CD003262.pub5 PMC6481562

[24] Wladis EJ , Bradley EA , Bilyk JR , Yen MT , Mawn LA . Oral antibiotics for meibomian gland‐related ocular surface disease: a report by the American academy of ophthalmology. Ophthalmology. 2016;123(3):492–496. 10.1016/j.ophtha.2015.10.062 26707417

[25] Wladis EJ , Adam AP . Immune signaling in rosacea. Ocul Surf. 2021;22:224–229. 10.1016/j.jtos.2021.08.017 34481075

[26] Wladis EJ , Carlson JA , Wang MS , Bhoiwala DP , Adam AP . Toll‐like receptors and vascular markers in ocular rosacea. Ophthalmic Plast Reconstr Surg. 2013;29(4):290–293. 10.1097/iop.0b013e318293764c 23839635

[27] Yamasaki K , Kanada K , Macleod DT , Borkowski AW , Morizane S , Nakatsuji T , et al. TLR2 expression is increased in rosacea and stimulates enhanced serine protease production by keratinocytes. J Invest Dermatol. 2011;131(3):688–697. 10.1038/jid.2010.351 21107351 PMC3085277

[28] Yamasaki K , Di Nardo A , Bardan A , Murakami M , Ohtake T , Coda A , et al. Increased serine protease activity and cathelicidin promotes skin inflammation in rosacea. Nat Med. 2007;13(8):975–980. 10.1038/nm1616 17676051

[29] Wladis EJ , Lau KW , Adam AP . Nuclear factor Kappa‐B is enriched in eyelid specimens of rosacea: implications for pathogenesis and therapy. Am J Ophthalmol. 2019;201:72–81. 10.1016/j.ajo.2019.01.018 30703356

[30] Wladis EJ , Arunachalam T , LaJoie JE , Lau KW , Adam AP . Myeloid differentiation factor 88 expression in eyelid specimens of rosacea. Orbit. 2021;41(3):1–6. 10.1080/01676830.2021.1905668 33789561

[31] Aldrich N , Gerstenblith M , Fu P , Tuttle MS , Varma P , Gotow E , et al. Genetic vs environmental factors that correlate with rosacea: a cohort‐based survey of twins. JAMA Dermatol. 2015;151(11):1213–1219. 10.1001/jamadermatol.2015.2230 26307938

[32] Aponte JL , Chiano MN , Yerges‐Armstrong LM , Hinds DA , Tian C , Gupta A , et al. Assessment of rosacea symptom severity by genome‐wide association study and expression analysis highlights immuno‐inflammatory and skin pigmentation genes. Hum Mol Genet. 2018;27(15):2762–2772. 10.1093/hmg/ddy184 29771307 PMC6822543

[33] Kim C , Giaccone G . MEK inhibitors under development for treatment of non‐small‐cell lung cancer. Expet Opin Invest Drugs. 2018;27(1):17–30. 10.1080/13543784.2018.1415324 29216787

[34] Lian T , Li C , Wang H . Trametinib in the treatment of multiple malignancies harboring MEK1 mutations. Cancer Treat Rev. 2019;81:101907. 10.1016/j.ctrv.2019.101907 31715422

[35] Wang D , Ge L , Guo Z , Li Y , Zhu B , Wang W , et al. Efficacy and safety of trametinib in neurofibromatosis type 1‐associated plexiform neurofibroma and low‐grade glioma: a systematic review and meta‐analysis. Pharmaceuticals. 2022;15(8):956. 10.3390/ph15080956 36015104 PMC9415905

[36] Hilbring C , Augustin M , Kirsten N , Mohr N . Epidemiology of rosacea in a population‐based study of 161,269 German employees. Int J Dermatol. 2022;61(5):570–576. 10.1111/ijd.15989 34897653

[37] Rainer BM , Kang S , Chien AL . Rosacea: epidemiology, pathogenesis, and treatment. Derm Endocrinol. 2017;9(1):e1361574. 10.1080/19381980.2017.1361574 PMC5821167 29484096

[38] Braunholtz DA , Edwards SJL , Lilford RJ . Are randomized clinical trials good for us (in the short term)? Evidence for a "trial effect. J Clin Epidemiol. 2001;54(3):217–224. 10.1016/s0895-4356(00)00305-x 11223318

[39] McCarney R , Warner J , Iliffe S , van Haselen R , Griffin M , Fisher P . The Hawthorne Effect: a randomised, controlled trial. BMC Med Res Methodol. 2007;7(1):30. 10.1186/1471-2288-7-30 17608932 PMC1936999

[40] Wolfe F , Michaud K . The Hawthorne effect, sponsored trials, and the overestimation of treatment effectiveness. J Rheumatol. 2010;37(11):2216–2220. 10.3899/jrheum.100497 20843902

